# Acceptance and normalization of aromatherapy in emergency department rest areas: a mixed-methods study using Normalization Process Theory

**DOI:** 10.3389/fpubh.2026.1900791

**Published:** 2026-08-12

**Authors:** Jian Liu, Zhiqiang He, Xiuxia Yang, Fanglei Xu

**Affiliations:** 1 Department of Emergency, Tongji Hospital, Tongji University School of Medicine, Shanghai, China; 2 Department of Nursing, Tongji Hospital, Tongji University School of Medicine, Shanghai, China

**Keywords:** aromatherapy, emergency department, explanatory sequential design, Normalization Process Theory, nurses, path analysis, perceived barriers

## Abstract

**Objective:**

To examine factors associated with emergency department nurses' acceptance of aromatherapy interventions in rest areas and to explore the internal relationships among core mechanisms of Normalization Process Theory (NPT).

**Methods:**

An explanatory sequential mixed-methods design was employed. In Phase I, a cross-sectional survey was conducted among 601 emergency department nurses from 30 tertiary Grade A hospitals across multiple regions in China. An NPT-informed acceptance questionnaire was developed, and its structure was refined into a 19-item model using exploratory factor analysis (development sample, *n* = 300) and confirmatory factor analysis (validation sample, *n* = 301). Exploratory path analysis was then performed in the full sample (*n* = 601). In Phase II, 18 nurses were purposively selected based on quantitative findings for semi-structured interviews. Thematic analysis was used to contextualize and explain the quantitative results.

**Results:**

A total of 601 nurses were included. The mean acceptance score was 73.74 ± 12.07 (95% CI: 72.77–74.71), indicating a moderately high level of acceptance. In univariate analyses, educational level, job role, allergy history, chronic respiratory conditions or odor sensitivity, and odor-related discomfort in the past year were associated with acceptance (all *p* < 0.05). In multiple regression analysis, higher night shift frequency (β = −0.128, *p* = 0.003), absence of a definite allergy history (β = 0.118, *p* = 0.006), and no odor-related discomfort in the past year (β = 0.178, *p* < 0.001) remained independently associated with acceptance. Split-sample validation demonstrated that the revised 19-item model provided a better fit than the initial 23-item five-factor model and showed good fit in the full sample. Path analysis revealed theory-consistent positive relationships from coherence to cognitive participation and reflexive monitoring, which in turn predicted collective action. Perceived barriers were negatively associated with collective action (all *p* < 0.001). Model fit was acceptable.

**Conclusion:**

Emergency department nurses demonstrated a moderately high level of acceptance of aromatherapy interventions in rest areas. This study provides preliminary evidence on the interrelationships among core NPT mechanisms and identifies perceived barriers as an independent constraint on collective action. Given the cross-sectional design and the use of a single dataset for both model refinement and path analysis, these findings should be interpreted as exploratory. It is important to note that this study assessed acceptance as an implementation outcome and did not evaluate the clinical effectiveness of aromatherapy.

## 1 Introduction

The emergency department is a frontline setting for the management of critically ill patients. Its high-intensity, complex, and fast-paced work environment places substantial strain on nurses' occupational health, with potential consequences for workforce stability and patient safety. Burnout among nurses is widely recognized as a significant occupational health concern, with a substantial proportion of nurses experiencing burnout symptoms globally (1–3). This burden is particularly relevant in emergency department settings, where high workload and emotional demands may further increase vulnerability among nursing staff (4, 5).

Conventional interventions, including mindfulness training and psychoeducational programs, have shown benefit but are often difficult to sustain in the time-pressured emergency care environment (6–8). Aromatherapy was selected as a potential workplace intervention because it is non-invasive, relatively low-burden, and can be integrated into existing rest periods without substantially disrupting workflow. It has been associated in several systematic reviews with improvements in fatigue, anxiety, and sleep quality among nurses (9–11). Unlike many active or resource-intensive interventions, it allows flexible adjustment of scent, concentration, and duration to accommodate individual preferences and safety considerations. However, existing studies have mainly focused on effectiveness, while implementation-related acceptance remains insufficiently explored.

To address this gap, we drew on Normalization Process Theory (NPT) as our analytical framework. NPT identifies four core mechanisms: coherence, cognitive participation, collective action, and reflexive monitoring (12). Quantitative analyses of the relationships among these mechanisms in nursing implementation contexts remain limited. In addition, measurement instruments for NPT constructs tailored to this population and setting have yet to be fully validated and contextually adapted (13). Prior research, including work by Williams et al. (14), suggests that theory-informed implementation strategies can activate NPT mechanisms and support sustained use. However, the internal pathways linking these mechanisms have not been systematically examined. Implementation is also shaped by contextual constraints, such as perceived risks and organizational conditions. May et al. (15) noted that such constraints can directly inhibit collective action, independent of the core NPT mechanisms. Accordingly, a growing number of mixed-methods NPT studies have incorporated barriers as an auxiliary analytical dimension. To better reflect real-world settings, the present study includes perceived barriers as an extended contextual factor that may influence how core mechanisms translate into collective action.

Against this background, we applied NPT within an explanatory sequential mixed-methods design. The quantitative phase assessed acceptance of aromatherapy interventions in emergency department rest areas and examined associated factors, while exploratorily testing theory-informed pathways among NPT constructs. The qualitative phase then provided contextual interpretation of these relationships, with particular attention to implementation processes and constraints. This study aims to generate practice-oriented evidence to inform the integration and sustained use of aromatherapy interventions in emergency department settings.

## 2 Methods

### 2.1 Study design

This study used an explanatory sequential mixed methods design (16). Phase I comprised a cross-sectional survey. Based on item performance, the measurement model was refined, and nurses' acceptance of aromatherapy interventions, along with associated factors, was quantified. Using the same dataset, exploratory path analysis was conducted in the full sample to examine associations among the core dimensions of NPT and to assess the relationship between perceived barriers, specified as an extended contextual factor, and collective action (17). Because model refinement (exploratory factor analysis [EFA] and confirmatory factor analysis [CFA]) and path analysis were performed on the same dataset (*n* = 601), the structural findings are interpreted as exploratory to reduce the risk of overfitting. Phase II consisted of semi-structured interviews. Participants were purposively selected based on Phase I results, and interviews were conducted to provide an in-depth understanding of the mechanisms underlying the quantitative findings.

After completing the thematic analysis, we integrated the quantitative and qualitative findings by systematically comparing regression and path coefficients with qualitative themes and representative quotations. For each comparison, team members independently classified the qualitative evidence as reinforcing or explaining, deepening or supplementing, or contradicting the quantitative findings; discrepancies were resolved through discussion (Table 1).

**Table 1:** Joint display of integrated quantitative and qualitative findings.

Key quantitative findings	Corresponding qualitative themes/subthemes	Representative quotations	Consistency assessment
Higher night shift frequency → lower acceptance (*p* = 0.003)	Perceived barriers/Concerns about workload and token implementation; Cognitive participation/Differentiated willingness to participate and information engagement.	“We can try it, but make it not too complicated.” “I won't actively seek it out; I'll just glance at it if I come across it.”	Consistent–explains/reinforces
No allergy history → higher acceptance (*p* = 0.006)	Perceived barriers/Health safety and professional image concerns.	“My biggest concern is my own health. An asthma attack is no joke.”	Consistent–explains/reinforces
No odor-related discomfort → higher acceptance (*p* < 0.001)	Perceived barriers/Health safety and professional image concerns; Coherence/Meaning construction of restorative value and employee care.	“Patients might think we are unprofessional.”	Consistent–explains/reinforces
Coherence → Cognitive participation → Reflexive monitoring → Collective action (all *p* < 0.001)	Reflexive monitoring/Pilot-based evaluation and convenient anonymous feedback; Reflexive monitoring/Pragmatic orientation toward dynamic optimization and termination decisions.	“Start with one shift or one area as a trial… if it works well, then expand it.” “If it does not work well, then stop it. There is no need to continue just for the sake of doing it.”	Consistent
Perceived barriers → Collective action ↓ (*p* < 0.001)	Perceived barriers/Concerns about workload and token implementation.	“My biggest worry is ‘3-min enthusiasm.' Our department has had too many projects that started strong but ended weak.”	Consistent–explains/reinforces
Nurses with allergy history showed higher participation motivation in qualitative findings	Cognitive participation/Participation confidence driven by organizational support and risk protection.	“Precisely because I have allergies, I hope this can be implemented in a scientific way.”	Deepens/supplements

### 2.2 Participants

This study was approved by the Ethics Committee of Shanghai Tongji Hospital (Approval No. K-2026-019). The questionnaire landing page outlined the study purpose, anonymity, voluntary participation, and the right to withdraw. Participants proceeded after providing informed consent electronically, and submission of the questionnaire was taken as consent. Data was collected between January and March 2026, with the formal survey conducted from February to March following ethical approval. Using convenience sampling, 601 emergency department nurses were recruited from 30 tertiary Grade A hospitals across multiple regions in China. Inclusion criteria were: (1) possession of a valid nursing license; (2) at least 1 year of experience in emergency nursing; and (3) willingness to participate. Exclusion criteria were: (1) interns, trainees, or visiting nurses; (2) continuous leave exceeding 1 month for any reason; and (3) refusal to participate.

### 2.3 Study instruments

#### 2.3.1 General information questionnaire

A structured questionnaire was developed based on prior literature (18–21). It collected demographic and occupational information, including sex, age, professional title, years of experience, primary job role, night shift frequency, and allergy history.

#### 2.3.2 NPT-based questionnaire on acceptance of aromatherapy implementation in emergency department rest areas

This instrument was developed using the NPT framework and relevant literature. Items corresponding to the four core NPT mechanisms were generated with reference to the Chinese version of the NoMAD (Normalization MeAsure Development questionnaire) (22). Additional items were included to capture perceived barriers specific to the implementation of aromatherapy in emergency department rest areas. These items assessed constraints related to safety, workload, resource availability, and professional boundaries. In this study, perceived barriers were conceptualized as an extended contextual construct, distinct from the four core NPT mechanisms and closely tied to the implementation setting. In the exploratory path analysis, this construct was specified as an exogenous variable to examine its direct association with collective action.

The Delphi method (23, 24) was used to establish the content validity of the questionnaire through two rounds of expert consultation. Twelve experts participated. Response rates were 100.00% in the first round and 91.67% in the second. All experts held at least intermediate professional titles and had backgrounds in nursing management, emergency nursing, clinical research, or related fields. The mean age of the panel was 39.33 ± 3.31 years, with a mean of 13.33 ± 2.15 years of professional experience. An item-level content validity index (I-CVI) ≥ 0.78 and a scale-level average content validity index (S-CVI/Ave) ≥0.90 were considered acceptable (25–27). The questionnaire demonstrated strong content validity (I-CVI = 0.833–1.000; S-CVI/Ave = 0.994).

The final questionnaire comprised six sections and 39 items, including 23 items assessing core constructs. Following EFA and CFA, four poorly performing items were removed, yielding a revised 19-item model used in all subsequent analyses. Items were scored on a 5-point Likert scale (1 = strongly disagree to 5 = strongly agree), giving a total score range of 19–95. Based on quartile thresholds, scores were categorized as low (≤66), moderate (67–82), or high (≥83) acceptance levels. Higher scores indicate greater acceptance of aromatherapy interventions in the emergency department rest areas.

Prior to the formal survey, a pilot study was conducted with 50 emergency department nurses from tertiary Grade A hospitals not included in the main sample. This pilot, conducted before ethical approval, was used solely to assess clarity, wording, and procedural feasibility and was not included in the final analysis. Internal consistency was high, with an overall Cronbach's α of 0.934 and dimension-specific α values of 0.936, 0.858, 0.865, and 0.913.

#### 2.3.3 Data collection

##### 2.3.3.1 Quantitative data

**Figure 1:**
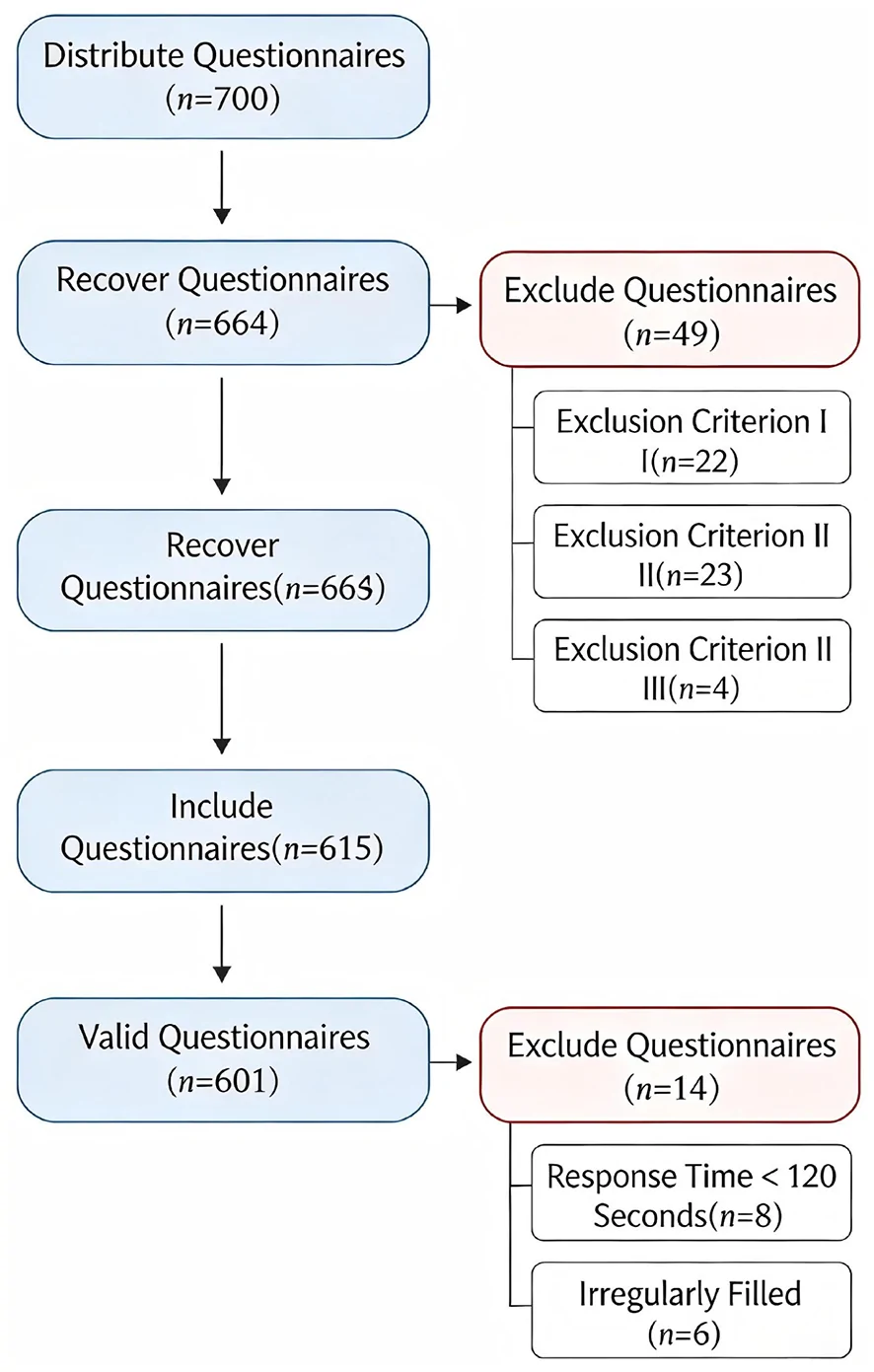
Questionnaire Screening Flowchart.

Data were collected using an online survey platform (Wenjuanxing). Skip logic and mandatory-response settings were applied to minimize missing data. After obtaining permission from nursing administrators and department heads, the questionnaire was distributed within emergency departments. Nurses completed the survey anonymously online. Data collection took place from February to March 2026. Of 700 questionnaires distributed, 664 were returned. Responses completed in less than 120 s, with evident response patterns, or otherwise deemed invalid, were excluded, yielding 601 valid questionnaires (effective response rate: 85.86%; see Figure 1). In the full sample, the overall Cronbach's α was 0.931. The α coefficients for the four dimensions were 0.702, 0.742, 0.752, and 0.803, respectively. Although slightly lower than in the pilot study, likely reflecting the larger sample size and reduction in item number (from 23 to 19), all values met the conventional threshold of 0.70, indicating acceptable internal consistency.

Sample size was estimated using G^*^Power 3.1.9.7. The following parameters were specified: effect size *f*^2^ = 0.15 (medium, based on Jacob Cohen, 1988), significance level α = 0.05, statistical power (1-β) = 0.80, and 23 predictors. The minimum required sample size was 501. The final sample (*n* = 601) exceeded this threshold, indicating adequate statistical power.

##### 2.3.3.2 Qualitative data

Following completion of the quantitative analysis, participants were purposively selected for semi-structured interviews to capture a range of perspectives. Sampling was guided by the principle of theoretical saturation and informed by key quantitative characteristics: (1) highest acceptance scores, (2) lowest acceptance scores, (3) presence of a definite allergy history, and (4) absence of an allergy history. An interview guide was developed based on the four core constructs of NPT. With participants' consent, interviews were audio-recorded and transcribed verbatim. All data were anonymized and stored in encrypted files accessible only to the research team. Recruitment continued until thematic saturation was reached, defined as the point at which no new themes emerged in three consecutive interviews. In total, 18 nurses were interviewed.

### 2.4 Statistical methods

#### 2.4.1 Quantitative data

Data analysis was conducted using SPSS Statistics 26.0 (IBM Corp., Armonk, NY, USA), R version 3.4.3 (R Foundation, Vienna, Austria), and AMOS 28.0 (IBM Corp., Armonk, NY, USA). Normality was assessed using the Shapiro–Wilk test. Continuous variables with normal distributions are presented as mean ± standard deviation, whereas non-normally distributed variables are reported as median and interquartile range. Group differences were examined using *t*-tests or analysis of variance, as appropriate. Categorical variables are presented as frequencies and percentages.

Internal consistency of the questionnaire was evaluated using Cronbach's α, with values ≥0.70 considered acceptable. Structural validity was assessed through a split-sample approach, with EFA conducted in the development sample (*n* = 300) and CFA in the validation sample (*n* = 301). EFA included the Kaiser–Meyer–Olkin (KMO) test, Bartlett's test of sphericity, parallel analysis, and Promax oblique rotation. Model fit in CFA was evaluated using standard indices: χ^2^/df < 3 indicated good fit and < 5 acceptable fit; root mean square error of approximation (RMSEA) < 0.08 indicated acceptable fit; comparative fit index (CFI) >0.90 indicated good fit; and standardized root mean square residual (SRMR) < 0.08 indicated acceptable fit (28). Perceived barriers were specified as an exogenous contextual variable with a direct effect on collective action. This specification was theoretically driven: perceived barriers were conceptualized as external constraints specific to the emergency department aromatherapy context, rather than a core NPT mechanism. Alternative structural roles (e.g., mediating or moderating roles) are theoretically plausible but could not be rigorously evaluated given the cross-sectional design; these possibilities should be examined in future longitudinal studies or independent samples.

Given the exploratory nature of the path analysis, and the fact that both measurement refinement and structural estimation were conducted using the same dataset, we did not perform bootstrapping or k-fold cross-validation. Such resampling approaches would provide only within-sample variability and cannot substitute for external validation. Accordingly, we report unadjusted parameter estimates and their consistency with theory, and emphasize that confirmatory testing in independent samples is a priority for future research.

One-way analysis of variance (ANOVA) was used to compare overall acceptance and scores across NPT dimensions among nurses with different demographic characteristics, and to identify candidate variables associated with acceptance. To determine independent factors, overall acceptance was specified as the dependent variable. Variables that were statistically significant in the ANOVA or considered theoretically relevant were entered into a multiple linear regression model. Unstandardized coefficients (*B*), standardized coefficients (β), and 95% confidence intervals were reported. Multicollinearity was assessed using variance inflation factors (VIF), with values < 5 indicating no substantial collinearity. It should be noted that overall acceptance was entered as a continuous score in all inferential analyses and was not discretized.

Following scale refinement, exploratory path analysis (29) was conducted in the full sample (*n* = 601) using the revised 19-item model. This analysis examined the relationships among the four core constructs of NPT and assessed the association between perceived barriers, specified as an extended contextual factor, and collective action. Parameters were estimated using the maximum likelihood method. The prespecified model followed a theoretically informed sequence: coherence → cognitive participation → reflexive monitoring → collective action. Because the study did not include an independent validation sample for hypothesis testing and did not model perceived barriers as a moderator or mediator, this variable was treated as an external contextual factor rather than a core NPT mechanism. It was assumed to relate most directly to the action stage (collective action). Accordingly, perceived barriers are not interpreted here as a fifth NPT construct or as a confirmed moderating factor. Given the cross-sectional design, the path analysis is interpreted as exploratory and intended primarily to assess theoretical plausibility rather than to support causal inference. Validation in independent samples is therefore warranted.

#### 2.4.2 Qualitative data

A theory-driven thematic analysis based on the NPT framework was conducted using NVivo 12.0 (30). Two researchers independently read and coded the transcripts. Discrepancies were resolved through discussion with reference to the original data; if consensus could not be reached, a third senior researcher adjudicated. Themes were developed iteratively through constant comparison and inductive analysis, then reviewed and refined through team discussion. Data collection continued until no new themes emerged, indicating theoretical saturation. Analytical rigor was supported through member checking and peer debriefing. The final thematic framework was organized around the four core NPT constructs, with perceived barriers presented as an overarching contextual theme.

## 3 Results

### 3.1 Demographic and work-related characteristics of the participants

A total of 601 emergency department nurses completed the survey. Of the 601 participants, the majority were female (*n* = 437, 72.71%), aged 26–35 years (*n* = 311, 51.75%), had ≥10 years of emergency department experience (*n* = 283, 47.09%), worked primarily in the resuscitation room (*n* = 258, 42.93%), held a staff nurse title (*n* = 288, 47.92%), and had a bachelor's degree (*n* = 483, 80.37%). Regarding night shifts, 353 nurses (58.74%) reported working five or more-night shifts per month over the past 6 months. In addition, 94 nurses (15.64%) reported a confirmed history of allergies, 192 (31.95%) had chronic respiratory conditions or odor sensitivity, and 191 (31.78%) had experienced headaches or nausea triggered by strong odors in the past year. Detailed demographic and occupational characteristics are presented in Table 2.

**Table 2:** Demographic and work-related characteristics of the participants.

Item	Category	Frequency	Percentage (%)
1. Gender	Male	164	27.29
	Female	437	72.71
2. Age	≤25 years	58	9.65
	26 ~ 35 years	311	51.75
	36 ~ 45 years	193	32.11
	≥46 years	39	6.49
3. Total years of working in the emergency department	<1 year	32	5.32
	1–5 years	155	25.79
	6–10 years	131	21.80
	≥10 years	283	47.09
4. Current professional title	Nurse	81	13.48
	Senior nurse	288	47.92
	Nurse in charge	219	36.44
	Associate chief nurse and above	13	2.16
5. Highest educational level	Secondary vocational education	4	0.67
	Junior college	98	16.31
	Bachelor's degree	483	80.37
	Master's degree and above	16	2.66
6. Average number of night shifts per month in the past 6 months (including early- and late-night shifts)	None	74	12.31
	1–2 per month	48	7.99
	3–4 per month	126	20.97
	≥5 per month	353	58.74
7. Main work position	Triage desk	82	13.64
	Resuscitation room	258	42.93
	Observation room	69	11.48
	Treatment area (infusion/injection/wound care room)	115	19.13
	Other	77	12.81
8. History of allergies (especially to pollen, perfume, etc.)	Yes	94	15.64
	No	507	84.36
9. Chronic respiratory problems (such as asthma, allergic rhinitis) or high sensitivity to odors	Yes	192	31.95
	No	409	68.05
10. Experience of headache or nausea due to strong odors (such as strong perfume or disinfectant) in the past year	Yes	191	31.78
	No	410	68.22
Total		601	100

### 3.2 Questionnaire reliability and validity

Exploratory factor analysis (EFA) conducted in the development sample (*n* = 300) indicated that the data were suitable for factor analysis (KMO = 0.932; Bartlett's χ^2^ = 7,150.084, *p* < 0.001). Parallel analysis supported a four-factor solution, consistent with the four-core constructs of NPT. However, as parallel analysis is exploratory, we also tested a theory-driven five-factor model in which perceived barriers were specified as a distinct contextual construct (e.g., safety, resources, workload) separate from the four NPT mechanisms. Had the five-factor model demonstrated poor loadings or substantial overlap, this would have supported retention of the four-factor solution.

The initial 23-item extended five-factor model showed poor fit in the validation sample (*n* = 301): χ^2^/df = 4.419, CFI = 0.903, TLI = 0.889, RMSEA = 0.107 (90% CI: 0.100–0.114), and SRMR = 0.068, indicating that the need for model revision. Based on EFA results and standardized factor loadings, four poorly performing items were removed, yielding a revised 19-item model. Items were retained if their standardized factor loading ≥0.40. Specifically, two items from the cognitive participation dimension (both with squared multiple correlations [SMC] of 0.005) and two items from the perceived barriers dimension (SMC = 0.047 and 0.035) were excluded. In the revised model, the standardized factor loadings ranged from 0.809 to 0.962.

The revised model demonstrated marginal fit in the validation sample: χ^2^/df = 3.592, CFI = 0.950, TLI = 0.940, RMSEA = 0.093, SRMR = 0.041. Model fit improved in the full sample (*n* = 601): χ^2^/df = 4.257, CFI = 0.968, TLI = 0.961, RMSEA = 0.074, SRMR = 0.029. Overall, the revised 19-item model showed improved stability but should be interpreted as demonstrating marginal to acceptable fit. Because the development and validation samples were drawn from the same population and later combined, these analyses are intended to assess theoretical coherence rather than to provide strict validation.

Based on the CFA results of the revised 19-item model, convergent and discriminant validity were further evaluated. Composite reliability (CR) values ranged from 0.901 to 0.972 across all dimensions, exceeding the recommended threshold of 0.70. Average variance extracted (AVE) values ranged from 0.740 to 0.885, all above 0.50, indicating good convergent validity. Discriminant validity was supported, as the square root of the AVE for each construct exceeded the inter-factor correlations, satisfying the Fornell-Larcker criterion.

Reliability analysis showed strong internal consistency, with an overall Cronbach's α of 0.930 and factor-specific α values ranging from 0.899 to 0.972. The perceived barriers construct was identified as an extended empirical factor derived from the implementation context of aromatherapy in emergency department rest areas, rather than a core component of NPT. This factor was reverse coded prior to inclusion in the structural model. Its emergence suggests that nurses' acceptance is shaped not only by core NPT mechanisms (coherence, cognitive participation, collective action, and reflexive monitoring), but also by contextual constraints such as safety concerns, resource availability, and perceived workload.

The relatively high standardized factor loadings (0.809–0.962) can be interpreted in several ways. First, the four NPT mechanisms are theoretically interrelated, so some shared variance is expected. Second, discriminant validity was supported empirically, as the square root of AVE exceeded inter-factor correlations (Fornell-Larcker criterion). Third, qualitative findings further supported the distinctiveness of the constructs. Taken together, the high loadings likely reflect strong explanatory power rather than a methodological artifact.

### 3.3 Current status of emergency department nurses' acceptance of aromatherapy interventions in rest areas

Normality testing (Shapiro–Wilk) indicated that scores for all dimensions deviated from a normal distribution (all *p* < 0.001). However, given that the absolute values of skewness and kurtosis were within acceptable ranges (|skewness| < 3, |kurtosis| < 10) (31), the data were treated as approximately normally distributed and are presented as mean ± standard deviation. Based on the revised 19-item model, the mean overall acceptance score was 73.74 ± 12.07 (95% CI: 72.77–74.71), indicating a moderate level of acceptance. Mean scores for the core dimensions of NPT were as follows: coherence, 20.94 ± 4.14 (95% CI: 20.61–21.27); cognitive participation, 8.27 ± 1.74 (95% CI: 8.13–8.41); collective action, 15.77 ± 3.19 (95% CI: 15.51–16.02); and reflexive monitoring, 20.58 ± 3.73 (95% CI: 20.28–20.87). Perceived barriers, included as an extended empirical dimension, had a reverse-scored mean of 8.19 ± 3.13 (95% CI: 7.94–8.44) and were analyzed separately in subsequent structural models. To maintain conceptual clarity, all descriptive statistics and inferential analyses reported here are based on the revised 19-item model and focus on the four core NPT constructs.

#### 3.3.1 One-way analysis of variance by participant characteristics

**Figure 2:**
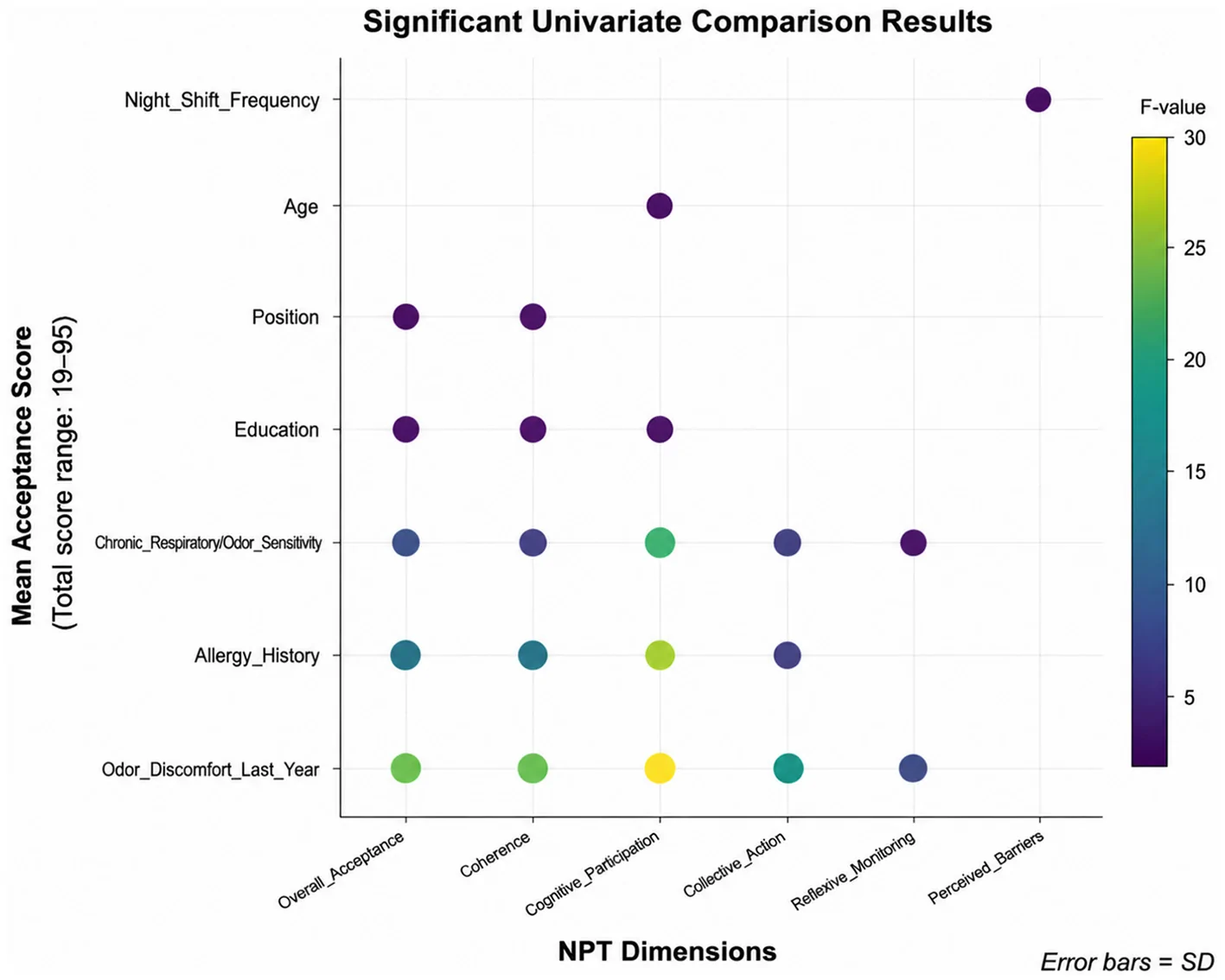
Main significant results of the one-way comparisons.

One-way ANOVA showed significant differences in overall acceptance across several characteristics: educational level (*F*(3, 597) = 3.300, *p* = 0.020, η*p*^2^ = 0.016), job post (*F*(4, 596) = 2.437, *p* = 0.046, ηp^2^ = 0.016), allergy history (F(1, 599) = 13.133, *p* < 0.001, ηp^2^ = 0.021), chronic respiratory conditions or odor sensitivity (*F*(1, 599) = 9.595, *p* = 0.002, ηp^2^ = 0.016), and odor-related discomfort in the past year (*F*(1, 599) = 24.924, *p* < 0.001, ηp^2^ = 0.040). Key pairwise differences across dimensions are illustrated in Figure 2.

#### 3.3.2 Multiple regression analysis of factors associated with acceptance

**Figure 3:**
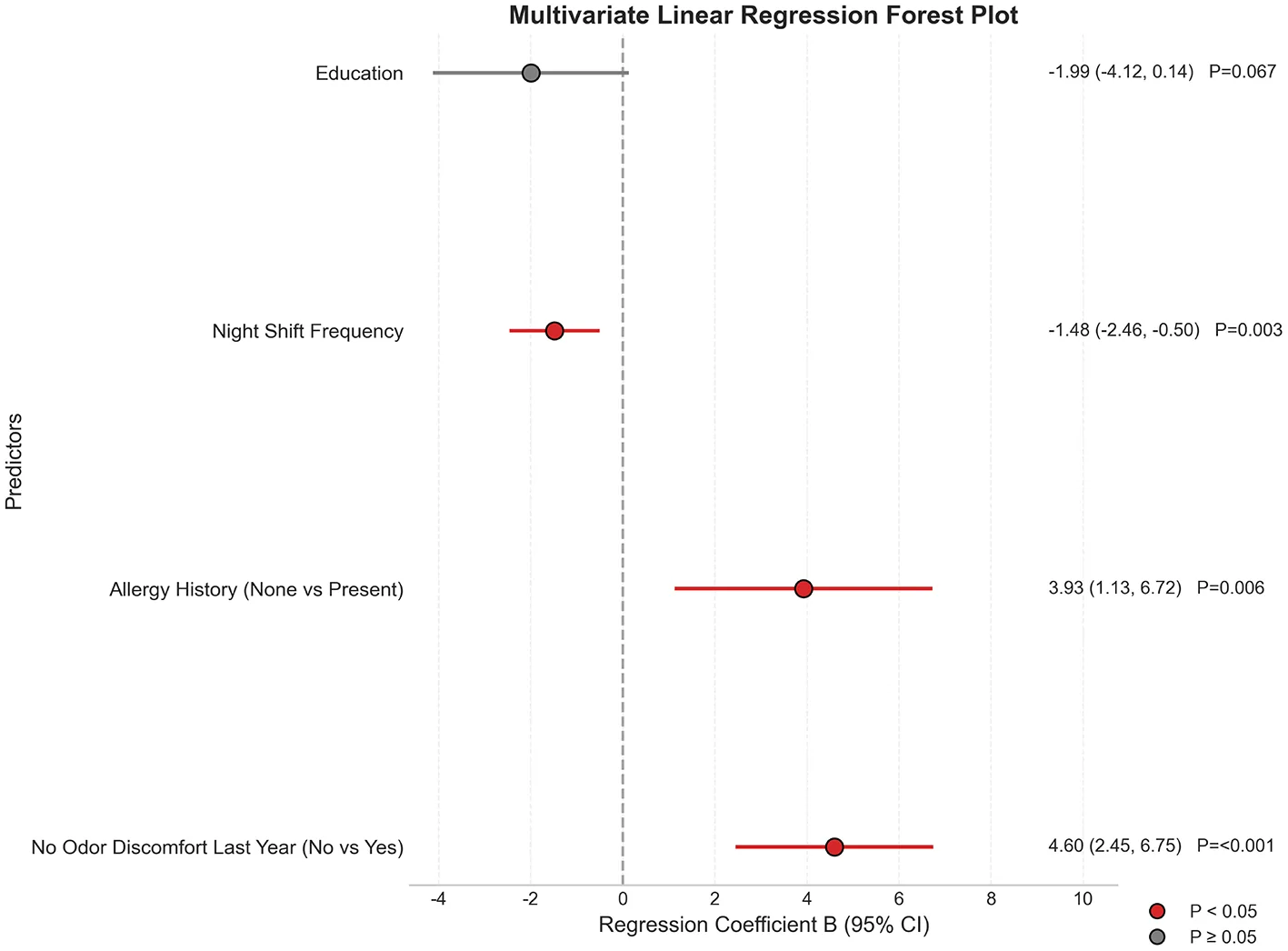
Forest plot of the multiple linear regression on factors influencing overall acceptance.

To identify independent predictors of acceptance, the total score from the revised 19-item model was entered as the dependent variable in a multiple linear regression model. Independent variables included sex, age, years of emergency department experience, professional title, educational level, night shift frequency, job post, allergy history, chronic respiratory conditions or odor sensitivity, and odor-related discomfort in the past year. The overall model was statistically significant (*F* = 5.013, *p* < 0.001), with an adjusted *R*^2^ of 0.074. Higher night shift frequency was associated with lower acceptance (*B* = −1.480, β = −0.128, 95% CI: −2.460 to −0.500, *p* = 0.003). In contrast, nurses without a definite allergy history (*B* = 3.929, β = 0.118, 95% CI: 1.133–6.724, *p* = 0.006) and those without odor-related discomfort in the past year (*B* = 4.599, β = 0.178, 95% CI: 2.449–6.749, *p* < 0.001) reported higher acceptance. Educational level showed a marginal negative association with acceptance (*B* = −1.991, β = −0.073, 95% CI: −4.119 to 0.138, *p* = 0.067), as illustrated in Figure 3. All variance inflation factors were below two, indicating no evidence of problematic multicollinearity.

#### 3.3.3 Exploratory path analysis: internal relationships among NPT mechanisms

After examining the influence of demographic and work-related factors on acceptance, we further explored the internal relationships among the four core constructs of NPT using the full sample (*n* = 601). The model included coherence, cognitive participation, reflexive monitoring, and collective action as core variables, with perceived barriers incorporated as an extended contextual factor in supplementary analyses. Because both the measurement model and path analysis were derived from the same dataset and the study used a cross-sectional design, the results should be interpreted cautiously. Specifically, they are intended to assess theoretical coherence rather than to provide precise effect estimates or support causal inference.

**Figure 4:**
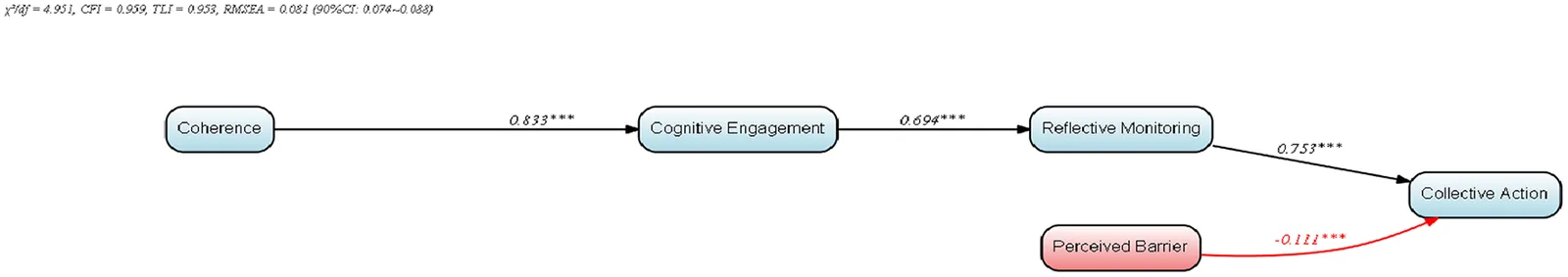
Final path model of NPT internal mechanisms and extended contextual factors. Path coefficients are standardized regression coefficients; ^***^indicates p < 0.001.

The prespecified model was supported. Coherence was positively associated with cognitive participation (β = 0.833, SE = 0.009, [95%CI: 0.331–0.367], *p* < 0.001), cognitive participation with reflexive monitoring (β = 0.694, SE = 0.063, [95% CI: 1.368–1.614], *p* < 0.001), and reflexive monitoring with collective action (β = 0.753, SE = 0.019, [95%CI: 0.070–0.144], *p* < 0.001). Perceived barriers showed a negative association with collective action (β = −0.111, SE = 0.022, [95%CI: −0.217 to −0.131], *p* < 0.001). Model fit indices were within acceptable ranges (χ^2^/df = 4.951, CFI = 0.959, TLI = 0.953, RMSEA = 0.081 [90% CI: 0.074–0.088], SRMR = 0.028; Table 3 and Figure 4). The final exploratory path model and standardized path coefficients are presented in Figure 4. Given the exploratory nature of the analysis, these indices should be interpreted as indicative rather than confirmatory. Overall, the findings suggest a coherent progression across NPT constructs: a clearer understanding of the intervention is associated with greater engagement, which in turn relates to more active evaluation and feedback, ultimately supporting collective implementation. In contrast, perceived barriers, particularly those related to safety, workload, and professional concerns, appear to constrain the translation of these processes into collective action. For the methodological reasons outlined in Section 1.4.1, specifically, that testing moderation effects requires independent samples to avoid overfitting, we did not examine the moderating role of perceived barriers in this exploratory phase. This issue is identified as a direction for future research.

**Table 3:** Path analysis results for NPT internal mechanisms and extended contextual factors.

Path	Standardized path coefficient	SE	CR	*p* value	95% CI
Coherence → Cognitive participation	0.833	0.009	36.845	<0.001	0.331–0.367
Cognitive participation → Reflexive monitoring	0.694	0.063	23.618	<0.001	1.368–1.614
Reflexive monitoring → Collective action	0.753	0.019	5.753	<0.001	0.070–0.144
Perceived barriers → Collective action	−0.111	0.022	7.877	<0.001	−0.217 to −0.131

#### 3.3.4 Implementation preferences among participants

Nurses most frequently preferred citrus scents (43.93%), followed by woody (25.46%) and floral (19.97%) fragrances. Ultrasonic aroma diffusers or humidifiers were the most commonly preferred delivery method (51.75%), followed by dedicated aroma diffusers (41.10%). Regarding timing, 59.90% of participants considered use during any rest period acceptable. A duration of 15–30 min per session was most commonly selected (53.24%). For scent persistence, “during rest periods only” was the most preferred option (37.44%). In terms of management preferences, 46.59% of respondents favored a centralized, department-level approach. These findings provide preliminary guidance for implementation. However, their associations with acceptance or with mechanisms from NPT were not systematically examined, as the primary aims of this study were to test relationships among NPT constructs and to identify independent predictors of acceptance. Future research could explore whether alignment between individual preferences and implementation strategies moderates' acceptance and related implementation outcomes.

### 3.4 Thematic analysis of acceptance formation and normalization process

#### 3.4.1 Characteristics of interview participants

To further interpret the quantitative findings, including levels of acceptance, associated factors, and underlying mechanisms, participants were selected using purposive sampling guided by theoretical saturation. A total of 18 emergency department nurses were interviewed. Their mean age was 34.83 ± 6.52 years; most were female (*n* = 13, 72.22%), held a bachelor's degree (*n* = 14, 77.78%), and reported frequent night shifts (*n* = 9, 50.00%).

#### 3.4.2 Thematic analysis based on the NPT framework

**Figure 5:**
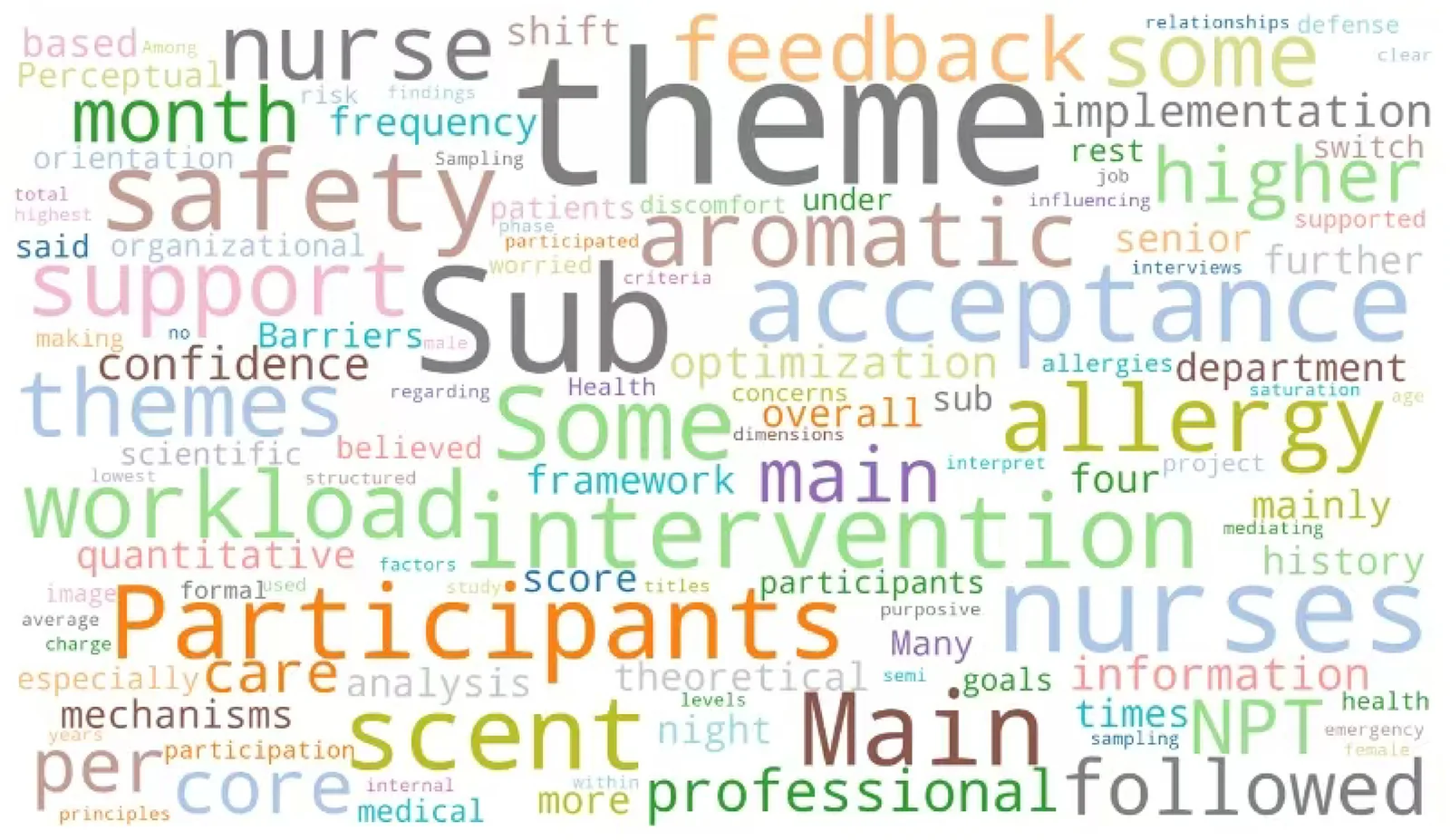
Hierarchical diagram of the interview data.

Interview data were analyzed using thematic analysis and coded deductively with reference to the NPT framework. The final analytical structure was organized around the four core NPT constructs (i.e., coherence, cognitive participation, collective action, and reflexive monitoring) with perceived barriers presented as an extended contextual theme (Figure 5). The initial coding process generated 43 codes, 19 subthemes, and five overarching themes. These were subsequently consolidated into five themes and 11 subthemes aligned with the four NPT constructs and the contextual factor of perceived barriers. The key findings, representative quotations, and corresponding questionnaire items for each NPT construct are presented in Table 4.

**Table 4:** Qualitative themes, representative quotations, and corresponding questionnaire items based on the Normalization Process Theory framework.

Theme	Subtheme	Key findings	Representative quotations	Representative questionnaire item
Coherence	Construction of meaning related to restorative value and employee care	Aromatherapy interventions were regarded as an “environmental switch” that facilitates transition from a high-pressure state to a restorative state, reflecting organizational support for nurses' psychological wellbeing.	“If there were a place where, once you enter, your body would know that it is time to rest… the scent is that switch.”	I understand the purpose of implementing aromatherapy in the rest area.
	Recognition of alignment with scientific evidence and clinical goals	Nurses with greater seniority or higher educational levels emphasized that the intervention should be supported by scientific evidence and aligned with patient safety and quality of care goals.	“When nurses are in a better state, they can be more patient with patients and reduce errors.”	
Cognitive participation	Differentiated willingness to participate and information engagement	Attitudes toward participation were categorized as active support, neutral trial, and avoidance, with information engagement primarily passive.	“It can be tried, but it should not be too complicated.” “I would not actively seek it out; I would just glance at it if I came across it.”	I am willing to participate in discussions regarding the implementation of aromatherapy in the department.
	Participation confidence driven by organizational support and risk protection	Genuine consideration of staff opinions and provision of resources by leadership enhanced confidence; nurses with allergy history participated in selection to ensure their own safety.	“Precisely because I have allergies, I hope this can be implemented in a scientific way.”	
Collective action	Division of responsibilities and demand for resource support	Strong opposition to increasing nurses' additional workload, with a preference for centralized management by logistics or administration and departmental provision of funding and equipment.	“It would be best if logistics or administration managed it centrally, with supervision by the head nurse.”	I believe that clear responsibilities should be assigned for managing aromatherapy equipment in the department.
	Consensus-based decision making and minority protection mechanisms	Support for forming consensus through voting or discussion, while emphasizing protection of the health rights of individuals with allergies or odor sensitivity.	“Priority should be given to protecting the most vulnerable individuals. If a scent can trigger asthma, then that scent should not be selected.”	
	Adaptation to physical environment and ventilation conditions	Physical conditions of the rest area, including space, ventilation, and storage, were key factors influencing implementation feasibility.	“The rest area is just a very small space… ventilation is a major issue and must be considered in advance.”	
Reflexive monitoring	Pilot-based evaluation and convenient anonymous feedback	Broad support for “pilot implementation for 1–3 months before wider rollout;” evaluation should be anonymous and digitalized, such as via QR codes, rather than paper-based.	“Start with one shift or one area as a trial… if it works well, then expand it.”	I believe that nurses' feedback on aromatherapy interventions should be collected regularly.
	Pragmatic orientation toward dynamic optimization and termination decisions	Support for adjusting scent type, concentration, and duration based on feedback; agreement to terminate the intervention if outcomes are unsatisfactory or if health issues arise.	“If it does not work well, then stop it. There is no need to continue just for the sake of doing it.”	
Perceived barriers	Concerns regarding health safety and professional image	Core concerns included allergies, respiratory discomfort, and equipment safety; scent residues on uniforms might lead to patient misunderstanding of professionalism.	“The biggest concern is my own health. An asthma attack is not trivial.” “Patients might think we are not professional.”	I am concerned that aromatherapy interventions may cause allergies or respiratory discomfort among colleagues.
	Concerns about workload and formalistic implementation	Concerns that the intervention may become short-lived or increase hidden workload, with insufficient sustained management.	“My biggest concern is that it will just be a short-lived initiative. Our department has had too many projects that started strong but ended weak.”	

Two qualitative patterns were particularly relevant to the quantitative findings. First, participants frequently described an incremental, “pilot-first” approach to implementation. One participant suggested, “Start with one shift or one area as a trial … if it works well, then expand it.” Another emphasized the conditional nature of continued engagement: “If it does not work well, then stop it. There is no need to continue just for the sake of doing it.” These accounts provide qualitative context for the theory-consistent associations observed among the NPT constructs, particularly the transition from engagement and appraisal to collective action.

Second, the negative association between perceived barriers and collective action identified in the exploratory path model was reinforced by concerns regarding sustainability, workload, and safety. One participant described a recurring concern: “My biggest worry is ‘3-min enthusiasm.' Our department has had too many projects that started strong but ended weak.” Health-related concerns were also prominent: “The biggest concern is my own health. An asthma attack is no joke.” These accounts suggest that perceived barriers may constrain the extent to which favorable understanding, engagement, and appraisal are translated into collective action.

Notably, the qualitative data also provided a more nuanced perspective on the quantitative finding that the absence of a definite allergy history was associated with higher acceptance. Although the regression analysis indicated lower acceptance scores among nurses with a definite allergy history at the group level, some interviewees with allergies did not reject the intervention outright. Instead, they expressed a safety-oriented and conditional willingness to participate. As one participant stated, “Precisely because I have allergies, I hope this can be implemented in a scientific way.” This finding suggests that prior sensitivity may not preclude engagement, but may make participation more dependent on clear safety protocols, individual choice, and transparent management. Thus, the qualitative evidence supplemented rather than directly contradicted the quantitative finding and demonstrated the added explanatory value of the mixed-methods design.

### 3.5 Qualitative interpretation of the quantitative findings

The qualitative findings were used to contextualize and interpret the quantitative results through triangulation. The observed negative association between night shift frequency and acceptance may reflect the effects of workload and fatigue, which can limit nurses' capacity to engage with new initiatives. Similarly, higher acceptance among nurses without a definite allergy history or recent odor-related discomfort is consistent with concerns about sensory tolerance. However, qualitative data suggests that prior sensitivity does not necessarily lead to rejection; rather, it heightens expectations regarding fragrance selection, concentration, device safety, and ventilation. A recurring theme in the interviews, “pilot first, gather feedback, and then refine,” provides a practical explanation for the sequential relationships observed among NPT constructs. Participants emphasized the importance of clear safety thresholds, defined responsibilities, and a manageable workload, which aligns with the positive progression from coherence to collective action observed in the quantitative model. At the same time, the negative association between perceived barriers and collective action is supported by qualitative accounts highlighting concerns about safety, resource availability, and operational burden (able 4). The identification of perceived barriers as an independent factor highlights the importance of contextual constraints in implementation. Although these factors do not modify the underlying conceptual structure of the four NPT mechanisms, they influence the extent to which these mechanisms are operationalized and enacted in practice.

## 4 Discussion

### 4.1 Acceptance of aromatherapy interventions in rest areas among emergency department nurses and influencing factors

This study found that emergency department nurses demonstrated a moderately high level of acceptance of aromatherapy interventions in rest areas. Acceptance was independently associated with night shift frequency, allergy history, and prior experiences of odor-related discomfort. A recent systematic review and meta-analysis found that aromatherapy is associated with reductions in stress, anxiety, and fatigue, as well as improvements in sleep quality among frontline hospital nurses, particularly in high-workload settings (9). Qualitative findings indicated that acceptance was not driven by fragrance preference *per se*, but rather by a broader appraisal of whether the intervention could support recovery, whether potential risks were manageable, and whether participation imposed minimal burden. Nurses with more frequent night shifts appeared less engaged with informational aspects of the intervention, likely reflecting fatigue-related constraints (32). In contrast, those with a history of allergies or odor sensitivity expressed heightened concerns regarding safety, including clearer boundaries, opt-out options, and autonomy in participation. Together, these findings suggest that perceived safety and low implementation burden are key determinants of acceptance in this setting. These findings reflect nurses' acceptance as an implementation outcome and should not be interpreted as evidence of aromatherapy's effectiveness on occupational health outcomes.

The standardized regression coefficients (β ≈ 0.1–0.18) and adjusted *R*^2^ (0.074) were modest, indicating that, although statistically significant, these factors accounted for a limited proportion of the variance in acceptance. This is consistent with expectations for complex, multidimensional psychosocial constructs. Importantly, small effect sizes are not necessarily trivial; even modest increases in acceptance may yield meaningful cumulative benefits in occupational health. Qualitative findings further indicated that nurses' support is contingent on multiple interacting conditions rather than any single determinant. Accordingly, the regression model should be interpreted as a tool to identifying higher-risk subgroups to inform targeted interventions, rather than as a comprehensive predictive model. Future research should incorporate a broader range of predictors, including organizational support, burnout, implementation climate, and leadership engagement, to develop a multi-level explanatory framework.

The transferability of these findings should be interpreted cautiously. Participants were recruited from tertiary Grade A hospitals in China, where staffing levels, organizational structures, institutional resources, and access to dedicated rest areas may differ from those in community hospitals or primary care settings. These contextual differences may alter both the feasibility of aromatherapy implementation and the relative importance of workload- and resource-related barriers. Previous hospital-based implementation studies have indicated that the integration of essential oil therapies depends on structured procedures, staff education, organizational support, and clearly defined safety practices (33, 34). Healthcare professionals' attitudes toward essential oils are also heterogeneous, with positive perceptions coexisting with concerns regarding evidence, safety, and appropriate use (35). Cultural and health-system differences in familiarity with complementary therapies, professional norms, individual autonomy, and risk management may therefore influence acceptance. Accordingly, the present findings may provide a preliminary reference for other settings, but direct transferability to community hospitals or international healthcare systems should not be assumed.

### 4.2 Structural adequacy of the revised 19-item model

Using a split-sample approach with EFA and CFA, the initial 23-item model was refined to a 19-item structure (24). The revised model demonstrated improved fit indices and internal consistency, indicating satisfactory structural stability within this population of emergency department nurses. These findings suggest that nurses' evaluations of aromatherapy interventions are shaped by multiple interrelated dimensions rather than a single attitudinal construct. The results also support the applicability of NPT measurement frameworks in this clinical context. However, because the EFA and CFA were conducted within the same overall sample, the potential for overfitting cannot be excluded. Accordingly, model fit indices and parameter estimates should be interpreted as preliminary. Replication and validation in independent samples are required to establish the robustness and generalizability of the model.

Although item reduction improved model fit, it may raise concerns regarding content validity. However, the four removed items exhibited very low SMCs, indicating minimal contribution to construct measurement; retaining them would likely have introduced noise rather than enhanced validity. Their removal did not result in conceptual gaps, as the relevant subdomains remained adequately represented. In addition, the Delphi process and expert team review provided independent support for content validity. Thus, the observed in model fit reflects the elimination of psychometrically weak items rather than a loss of content representativeness.

### 4.3 Internal relationships within the NPT framework

Exploratory path analysis identified positive associations among the four core NPT mechanisms (coherence → cognitive participation → reflexive monitoring → collective action), consistent with theoretical expectations. These findings are consistent with a 2023 nurse-led aromatherapy program, which reported that implementation improved nurses' self-efficacy and engagement with non-pharmacological care (36). The qualitative data provided a coherent interpretation of this pattern, reflected in a progression from “understanding the intervention” to “engaging with it,” “evaluating its effects,” and ultimately “incorporating it into routine practice.” Given the cross-sectional design, these findings should be interpreted as theory-consistent associations rather than evidence of causality. The qualitative findings provide contextual interpretation of these associations but do not establish temporal precedence or causal mechanisms. Longitudinal or experimental studies are needed to establish temporal ordering and to test the directional assumptions implied by the NPT framework (36). The particularly strong path coefficient from coherence to cognitive participation (β = 0.833) likely reflects their close, sequential relationship in this specific context: nurses' understanding of the rationale for aromatherapy in rest areas appears to strongly shape their willingness to engage. However, because the analysis was exploratory and based on a single dataset, this estimate may have inflated; validation in independent samples is required for more precise effect estimation.

### 4.4 Theoretical positioning and practical significance of “perceived barriers” as an extended contextual factor

This study identified perceived barriers as an additional factor outside the four core mechanisms of NPT, with a significant negative association with collective action. While the core NPT constructs describe the general cognitive and social processes through which an intervention is understood, engaged with, and integrated into practice, perceived barriers capture context-specific constraints relevant to the implementation of aromatherapy in emergency department settings. Given that perceived barriers do not form part of the original NPT framework, we conceptualize them here as a contextual constraint operating within the clinical environment. Rather than modifying the relationships among the four core mechanisms, this factor appears to act directly on collective action, limiting the extent to which an intervention is enacted in practice. Qualitative findings indicate that nurses' primary concerns relate to health and safety, additional workload, and professional boundaries. These results suggest that in high-pressure settings such as emergency departments, sustained acceptance of an intervention depends not only on its perceived value, but also on whether it fits within existing workflows and remains within acceptable safety limits (37). Even when an intervention is viewed favorably in principle, concerns arising at the point of implementation may prevent it from being enacted. Theoretically, this highlights the importance of incorporating context-specific constraints when applying NPT in complex clinical environments. Future implementation research should identify and test such factors across different settings and interventions to better delineate the practical boundaries of NPT and enhance its explanatory utility. Alternative structural roles for perceived barriers also warrant consideration. For example, perceived barriers may indirectly inhibit collective action by attenuating reflexive monitoring (i.e., a mediation pathway), as suggested by qualitative accounts such as: “try it for a while, but stop immediately if any colleague feels unwell.” They may also function as moderators, acting as threshold conditions on the relationship between core mechanisms and collective action; as one nurse noted, “even if I think it makes sense, if the burden is too high, I would rather not do it.” These mechanisms remain theoretical. Given the cross-sectional design and the risk of overfitting, they were not formally tested in this study. Future research should examine these mediation and moderation hypotheses using independent longitudinal samples to clarify the structural role of perceived barriers within the NPT network.

### 4.5 Integration of quantitative and qualitative findings

The integration of quantitative and qualitative data yielded three consistent insights. First, acceptance of aromatherapy was not determined by fragrance preference alone, but by a combination of perceived meaning, acceptable risk, and manageable workload. Quantitative results showed that allergy history, prior odor-related discomfort, and higher night shift burden were associated with lower acceptance. Qualitative data further clarified that these concerns centered on safety thresholds, ventilation conditions, autonomy in opting out, and the potential for added tasks. Second, the four core NPT mechanisms demonstrated a theory-consistent sequential relationship, progressing from meaning-making (coherence) to engagement (cognitive participation), followed by appraisal (reflexive monitoring), and ultimately to implementation (collective action). This pattern aligns with participants' descriptions of a process of “first understanding, then trying, and finally evaluating the effects,” providing contextual support for the observed statistical associations. Third, perceived barriers functioned not as an additional NPT mechanism, but as a contextual threshold at the stage of action. When nurses considered issues of safety, resource demands, and professional boundaries to be manageable, earlier stages of understanding and engagement were more likely to be translated into practice. Conversely, even when coherence and participation were present, perceived constraints could still prevent implementation. Overall, these findings suggest that aromatherapy interventions in emergency department rest areas are best positioned as optional, low-burden environmental supports that can be introduced incrementally. In practice, implementation should prioritize voluntary participation, small-scale piloting, careful risk assessment, iterative refinement based on feedback, and clear opt-out mechanisms. Hospital administrators should also establish standardized procedures for fragrance selection, concentration, duration, ventilation, device maintenance, and staff responsibilities. Safety monitoring should include screening for allergy or respiratory sensitivity, documentation of adverse symptoms, and clear criteria for reducing or discontinuing exposure. Sustainability may be supported by assigning a coordinator and minimizing additional nursing workload. Although aromatherapy may require relatively limited resources, this study did not assess economic outcomes. Therefore, its cost-effectiveness should be evaluated in future implementation studies.

Beyond these insights, several unexpected findings suggest potential theoretical extensions of NPT. First, although regression indicated that the absence of allergy history was associated with higher acceptance, qualitative data revealed that nurses with a definite allergy history sometimes expressed stronger willingness to participate. This apparent inconsistency may reflect a form of a “self-protective participation motivation” within cognitive participation. Second, higher educational attainment and professional rank were marginally associated with lower acceptance, contrary to expectations. This pattern suggests that coherence may include a “depth of critical appraisal” dimension, whereby more highly trained nurses adopt a more cautious and systematic evaluation of interventions. Third, the direct negative effect of perceived barriers on collective action, combined with qualitative concerns about “3-min enthusiasm,” suggests a “situational action threshold,” a composite of perceived burden and safety concerns that may operate independently of NPT's sequential mechanisms and may interrupt the translation from cognition to action. Taken together, these findings suggest that applying NPT in high-pressure clinical settings may benefit from incorporating more nuanced constructs such as “risk-driven participation,” “depth of critical understanding,” and “situational action threshold” to enhance both explanatory and its explanatory capacity.

### 4.6 Limitations

Several limitations should be considered when interpreting the findings of this study. First, the revised 19-item model has not been validated in an independent external sample, and both the exploratory path analysis and model refinement were conducted using the same survey dataset. In addition, the cross-sectional design limits the ability to establish temporal ordering among variables. As such, the structural relationships identified should be regarded as exploratory and are not suitable for causal inference. Second, although perceived barriers were conceptualized as an extended contextual factor outside the four core NPT mechanisms, its precise structural role and its boundaries in relation to the core constructs remain unclear. Further research is needed to examine whether perceived barriers function as a moderator, mediator, or independent constraint within different implementation contexts. Third, this study focused on acceptance as the primary outcome and did not assess downstream outcomes such as actual adoption, sustained use, or occupational health effects. This limits the ability to determine whether acceptance translates into meaningful behavioral or clinical impact. Finally, the sample was drawn exclusively from emergency departments in tertiary Grade A hospitals in China, which may limit generalizability to community hospitals, primary care, or international settings. Convenience sampling may also have introduced selection bias. Future studies should validate our findings in diverse healthcare contexts and cross-cultural settings.

## 5 Conclusion

Drawing on NPT and an explanatory sequential mixed methods design, this study examined emergency department nurses' acceptance of aromatherapy interventions in rest areas and the underlying processes of their potential normalization. The findings indicate a moderately high level of overall acceptance, with night shift frequency, allergy history, and prior odor-related discomfort emerging as key influencing factors. The revised 19-item model demonstrated improved structural adequacy compared with the original 23-item version. Exploratory path analysis identified theory-consistent associations among coherence, cognitive participation, reflexive monitoring, and collective action. In addition, perceived barriers, conceptualized as an extended contextual factor, showed an independent negative association with collective action. Qualitative findings further supported a set of core conditions for acceptance, including understandable purpose, manageable risk, low burden, and the opportunity for feedback and adjustment. From a methodological perspective, this study contributes to ongoing efforts to operationalize NPT by providing preliminary quantitative evidence on the relationships among its core mechanisms and by examining the measurement properties of NPT-related constructs in a specific clinical population and implementation context. From a practical standpoint, the findings suggest that implementation strategies for aromatherapy interventions should emphasize clarity of purpose, safety assurance, minimal additional burden, and mechanisms for iterative feedback. Given the exploratory nature of the path analysis and the cross-sectional design, these findings should be considered hypothesis-generating rather than confirmatory. Future research should aim to replicate these findings in independent samples, examine the longitudinal relationships among NPT mechanisms, and further explore the role of perceived barriers in shaping implementation outcomes.

## Data Availability

The original contributions presented in the study are included in the article/supplementary material, further inquiries can be directed to the corresponding author.
